# Editorial for the Special Issue on Magnetic and Spin Devices

**DOI:** 10.3390/mi13040493

**Published:** 2022-03-22

**Authors:** Viktor Sverdlov, Nuttachai Jutong

**Affiliations:** 1 Christian Doppler Laboratory for Nonvolatile Magnetoresistive Memory and Logic, Institute for Microelectronics, TU Wien, 1040 Vienna, Austria; 2 Research Center for Quantum Technology, Faculty of Science, Chiang Mai University, Chiang Mai 50200, Thailand

As scaling of semiconductor devices displays signs of saturation, the focus of research in microelectronics shifts towards finding new computing paradigms based on novel physical principles. The electron spin is another intrinsic characteristic of an electron offering additional functionality to the electron charge-based semiconductor devices currently employed in microelectronics. Several fundamental issues including spin current injection, spin propagation, and relaxation, as well as spin orientation manipulation by the gate, have successfully been resolved enabling the electron spin for digital applications.

In order to generate and detect the spin-polarized currents by electrical means, magnetic metal contacts can be employed. The ferromagnetic contacts discussed by Boroš et al. [1,2] should be sufficiently small to comprise a single magnetic domain with a clear magnetization orientation. Magnetic moments of small domains were successfully used in the past and are still employed to store the information in magnetic hard disc drives. Herewith, the binary information is encoded into the domain’s magnetization orientation.

The magnetization of a domain creates a stray magnetic field which can be detected. Alternating magnetic moments create magnetic fields in opposite directions. Read heads can detect the field and read the information. It is shown by Khunkitti et al. [3] that magnetic heads with high sensitivity are important ingredients to enable ultrahigh areal densities of magnetic data storage technology. To write information, a magnetic field close to the magnetic domain is created by means of the electric currents running into the magnetic head. The recording density critically depends on the magnetic media properties as illustrated by Khunkitti et al. [4].

Without an external magnetic field, the magnetization of a magnetic domain is preserved and does not change over time. Therefore, adding magnetic domains in electronic devices introduces nonvolatility—the ability to preserve the functional state of a device without external power supplied. In addition, one can manipulate the magnetization orientation of a small magnetic domain by running a spin-polarized current through it. If the current is sufficiently strong, the magnetization of the domain aligns parallel with the spin current polarization. A purely electrical manipulation of a magnetic domain by electron currents provides an exciting opportunity to develop a conceptually new type of nonvolatile memories with improved scalability. The impinging spin-polarized current can be created by the charge current running through another ferromagnet separated from the small domain by a metallic spacer or a tunnel barrier. The electrical resistance of a sandwich made of two ferromagnetic contacts separated by a tunnel barrier (or a spacer) strongly depends on the relative magnetization orientation of the contacts in either parallel or anti-parallel configuration. Therefore, the binary information encoded into the relative magnetization is revealed through the sandwich’s resistance. This type of emerging memory is termed magnetoresistive. Magnetoresistive memories possess a simple structure. They offer excellent endurance and high speed of operation. Magnetoresistive memories are compatible with the metal–oxide–semiconductor field-effect transistor fabrication process. They open perspectives for conceptually new low power computing paradigms of data processing in the nonvolatile segment with the same devices used to store and to process the information.

Alternatively, spin currents can be generated by the spin Hall effect, when a pure spin current running normal to the interface of a heavy metal slab is produced by the charge current running through the slab. A torque due to the spin current acts on a ferromagnetic layer positioned on top of the heavy metal slab and may change the layer’s magnetization orientation. The size of the ferromagnetic layer determining the dimension of the memory cell can be significantly reduced if the magnetic layer is polarized perpendicularly to the interface of the ferromagnet layer with the slab; however, special care must be taken to deterministically switch the magnetization without an external magnetic field [5,6].

In line with most advanced spintronic devices, magnetic properties of ferromagnets are also exploited in a more traditional way as magnetic contacts in various security applications, ranging from access allowing magnetic cards to detectors in electrical security systems where the magnetic parts are installed on windows, door, or other objects whose change of state triggers an alarm as discussed by Boroš et al. [1]. The reliability parameters of the magnetic contact must be preserved within 10% variations regardless of extreme conditions of their functionality, which requires creation of a test environment allowing to stress the contacts under extreme conditions. Special experiments were designed and performed by Boroš et al. [1] in an air chamber with the temperature changing from −70 °C to +180 °C to investigate relations between the effect of temperature and the closing/opening distances for five selected types of magnetic contacts intended for outdoor use in. The automation of the testing process is essential to accelerate the reliability benchmarking and to eliminate human error. For this reason, the testing infrastructure described by Boroš et al. [2] was designed to use internal memory for evaluating the number of successful closures of magnetic contacts and then transmit the digitized data.

Current-perpendicular-to-the-plane giant magnetoresistance (CPP-GMR) sensors are promising as read heads for ultrahigh (> 1 Tb/in^2^) density magnetic data storages. The dependence of the free layer thickness on the stability of the Co_2_(Mn_0.6_Fe_0.4_)Ge Heusler-based CPP-GMR sensors was investigated by Khunkitti et al. [3]. The magnetoresistance ratio (MR), readback signal, dibit response asymmetry parameter, and power spectral density were calculated and characterized. It was demonstrated that the read head with a free layer thickness of 3 nm displays the best stability with a large MR of 26%.

To boost the density of magnetic storages even further, the magnetic media, as well as the data recording, must be improved. Exchange-coupled-composite-bit-patterned media (ECC-BPM) with microwave-assisted magnetic recording to improve the writability of the magnetic media at a 4 Tb/in^2^ recording density was proposed by Khunkitti et al. [4]. It was demonstrated that the switching field of the bilayer ECC-BPM is significantly reduced, with the lowest value of 12.2 kOe achieved at 5 GHz microwave frequency.

Spin-orbit torque magnetoresistive random access memory combines high-speed access with excellent endurance and is promising for application in caches. One of the options to achieve deterministic switching of a free layer with perpendicular magnetization without an external magnetic field is to use a two-pulse dynamic approach: the first pulse pre-selects the cell and puts the magnetization in-plane, while the second pulse completes the switching. As the switching probability depends on the amplitude and duration of the current pulses, finding an optimal sequence is paramount. A reinforcement learning approach in combination with micromagnetic simulations is introduced by de Orio et al. [5] to optimize the switching of a spin-orbit torque memory cell. The approach allows not only to find an optimal pulse sequence, but also to analyze the impact of material parameter variations on deterministic switching.

Field-free switching in perpendicular magnetic tunnel junctions is achieved by combing the spin-transfer (ST) and spin-orbit (SO) torques as demonstrated by Wasef and Fariborzi [6]. In particular, the relationship between the ST and SO critical current densities is obtained. The relationship between the critical SO and ST current densities depend not only on damping, but also on the magnitudes of the field-like torques opening the path towards designing magnetoresistive memories operated with help of both SO and STT currents.

In conclusion, we would like to thank all the authors for submitting their papers to this Special Issue. We also thank all the reviewers for dedicating their time and helping to improve the quality of the submitted papers. Last but not least, we would like to thank Ms. Aria Zeng from the *Micromachines* publishing office for her constant guidance and great support.
